# Synergistic Effect of Biomaterial and Stem Cell for Skin Tissue Engineering in Cutaneous Wound Healing: A Concise Review

**DOI:** 10.3390/polym13101546

**Published:** 2021-05-12

**Authors:** Shaima Maliha Riha, Manira Maarof, Mh Busra Fauzi

**Affiliations:** Centre for Tissue Engineering and Regenerative Medicine, Faculty of Medicine, Universiti Kebangsaan Malaysia, Kuala Lumpur 56000, Malaysia; shaimariha11@gmail.com (S.M.R.); manira@ppukm.ukm.edu.my (M.M.)

**Keywords:** wound healing, tissue engineering, skin regeneration, stem cells, natural/synthetic biomaterials

## Abstract

Skin tissue engineering has made remarkable progress in wound healing treatment with the advent of newer fabrication strategies using natural/synthetic polymers and stem cells. Stem cell therapy is used to treat a wide range of injuries and degenerative diseases of the skin. Nevertheless, many related studies demonstrated modest improvement in organ functions due to the low survival rate of transplanted cells at the targeted injured area. Thus, incorporating stem cells into biomaterial offer niches to transplanted stem cells, enhancing their delivery and therapeutic effects. Currently, through the skin tissue engineering approach, many attempts have employed biomaterials as a platform to improve the engraftment of implanted cells and facilitate the function of exogenous cells by mimicking the tissue microenvironment. This review aims to identify the limitations of stem cell therapy in wound healing treatment and potentially highlight how the use of various biomaterials can enhance the therapeutic efficiency of stem cells in tissue regeneration post-implantation. Moreover, the review discusses the combined effects of stem cells and biomaterials in in vitro and in vivo settings followed by identifying the key factors contributing to the treatment outcomes. Apart from stem cells and biomaterials, the role of growth factors and other cellular substitutes used in effective wound healing treatment has been mentioned. In conclusion, the synergistic effect of biomaterials and stem cells provided significant effectiveness in therapeutic outcomes mainly in wound healing improvement.

## 1. Introduction

The skin, an intricate structure composed of the epidermis, dermis, skin appendages, hair follicles, and sebaceous glands, is the body’s largest organ with direct exposure to the environment [1]. Healthy skin is essential in maintaining the human body’s physiological homeostasis as it protects the human body against infection, electrolyte loss, mechanical forces, and thermal imbalance. It also plays a pivotal role in dynamic processes such as hydration, initiation of vitamin D synthesis, and excretion [2]. Therefore, any disruption in skin integrity may lead to tissue disintegration, resulting in acute or chronic wounds. Acute wounds are traumatic injuries including burns or surgically created wounds that heal within an acceptable period of time, whereas chronic wounds including ulcers (diabetic, venous, pressure) or post-surgical wounds are those that fail to progress through the common healing process in a timely fashion, resulting in the lack of a significant recovery over a prolonged period of time [3].

The wound healing process comprises the coordination between the overlapping processes of inflammation, blood clotting, cellular proliferation, and extracellular matrix remodeling regulated by secretion of various growth factors, cytokines, and chemokines [1]. Malfunction in the following processes may lead to abnormal wound healing and failed regeneration leading to inconvenience in treatment, limiting wound repair and tissue integrity restoration. Numerous conventional and regenerative studies have been catered towards achieving effective wound therapies to reduce health costs and ensure successful scar healing and long-term relief [4]. However, regenerative therapy is preferred over the conventional one as it aims to restore skin function via re-establishing damaged cells and skin tissues without scarring. This therapeutic approach involves various strategies that include, but are not limited to, tissue engineering, stem cell transplantation, biomaterials, and growth factors therapy [2]. For instance, intravenous, intramuscular, and topical application of adipose-derived stem cells into mice with full-thickness wounds accelerated the healing rate along with improving wound closure [5].

In regenerative medicine, stem cell therapy has emerged as a new promising approach oriented towards wound healing as stem cells have the capacity of self-renewal and differentiation into multiple cell types, which is pivotal for tissue renewal and regeneration after an injury [6]. Although stem cell therapy has achieved improved healing via earlier wound closure and prevention of scar formation, it exhibits limitations in clinical applications due to poor survival and differentiation of the transplanted cells. Besides, factors such as the determination of the optimum cell source, the administration and processing of stem cells from the clinical standpoint, and the definition of the role of stem cells under precise clinical conditions have made the application of stem cell therapy challenging for regenerative wound healing [4]. To overcome these limitations, tissue engineering technology has emerged as an alternative to ensure improved viability and proliferative capacity of stem cells.

The emergence of skin tissue engineering has contributed to robust innovations in skin substitutes and replacement products for wound healing and tissue regeneration. The process includes various cells, biomaterials, biochemical, and physiochemical factors and engineering technologies to improve or replace skin tissues [2]. Growth factors, stem cells, and scaffolds are collectively known as the tissue engineering triad, and scientists have been looking for the best combination to use these tools to develop safer and cost-effective approaches for wound healing and repair [3]. The combination of stem cells with a specifically designed novel 3D biomaterial has different impacts on engineered skin post-implantation [6]. However, skin is inhabited by a plethora of cells arranged in a 3D matrix creating a complex microenvironment of cell–matrix interaction, making it difficult to mimic the native skin structure using conventional tissue engineering approaches [7]. Critical challenges in skin tissue engineering include the hierarchical complexity of skin anatomy, the compositional mismatch in terms of material properties, and the degradation rate of biological complications like varied cell numbers, cell types, matrix gradients in each layer, varied immune responses, and varied methods of fabrication [8]. In addition, with newer biomaterials being adopted for fabricating patient-specific skin substitutes and the emergence of stem cells, issues related to escalating processing costs, scalability, and stability of the constructs under in vivo conditions have raised some concerns [7].

Although various commercial substitutes are available presently, novel findings on fabrication techniques of biomaterials and regulators of wound healing have highly encouraged scientists to develop new engineered substitutes that offer an effective remedy for wound care and wound management [7]. The combination of stem cells or other cells with a specifically designed novel biomaterial has resulted in different impacts on engineered skin after wounding. An ideal biomaterial with multiple varieties of cultured cells and a collectively established broad knowledge of the healing process are the main criteria for the future development of skin substitutes [9]. While several natural and synthetic biomaterials and stem cells have been translated, only partial clinical success has been attained so far [4]. Hence, this review highlights the challenges of using stem cells alone in wound healing treatment and identifies key factors responsible for improved wound healing when stem cells are used in combination with biomaterials. Moreover, the review further focuses on the properties of biomaterials, their advantages, disadvantages, and application outcome and the role of growth factors and other cellular substitutes in various wound healing treatments. Furthermore, the research gap in skin tissue engineering in terms of stem cells and biomaterials used will be addressed.

## 2. Cutaneous Wounds

Wounds can be defined as injuries or any disorders in the typical structure of the skin caused by surgery, extrinsic factors (like pressure, burns, and cuts), or pathological conditions such as diabetes or vascular diseases [2]. Depending on the types, causes, and consequences of injuries, wounds can be clinically classified as an acute and chronic. Acute wounds are assorted injuries that destroy the integrity of soft tissue and proceed through an organized repair process within 4 to 6 weeks, resulting in the sustained restoration of anatomical and functional integrity [10]. Various mechanisms, trauma, or environmental exposures like extreme temperature changes, chemical contacts, radiation, or a microbial infection can give rise to acute wounds. Usually, acute wounds undergo repair processes in an orderly manner leading to a benign scar [10]. However, failure in the healing process due to wound area or depth can exceed the patient’s ability to heal, resulting in an undesirable scar, keloid formation, and a chronic or a non-healing wound [10]. Besides factors such as infection, physical agents, inflammation, and tumors can add to the chronic wound and delay the healing process for more than 12 weeks, preventing the damaged tissue from achieving its optimal and functional integrity [11].

**Figure 1:**
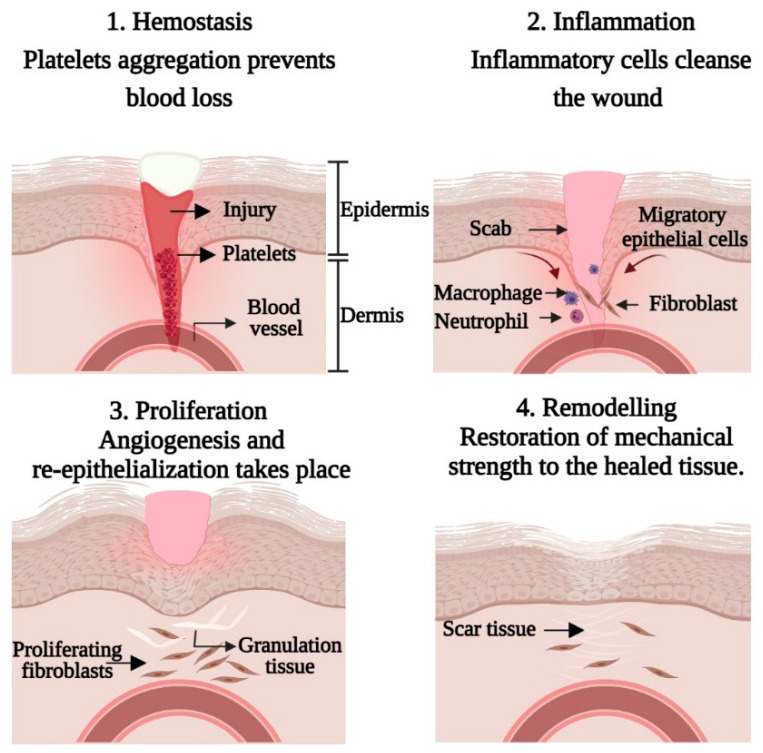
Regulated steps of wound healing (**1**) hemostasis; (**2**) inflammation; (**3**) proliferation and (**4**) remodeling of tissue (created with BioRender.com).

Dermal wound repair is a complex and dynamic process that requires interaction between dermal and epidermal cells, controlled angiogenesis, extracellular matrix, and plasma-derived proteins coordinated by cytokines and growth factors [6]. The healing action involves overlapping biological mechanisms and can be divided into three main regulated steps: Inflammation, proliferation, and remodeling (Figure 1).

The inflammation phase, which comprises the coagulation cascade, inflammatory pathway, and immune system involvement, occurs immediately after tissue damage to prevent infection, excessive loss of blood, fluids, and the removal of dead tissue [11]. During this phase, the generation of platelet clot helps achieve homeostasis, followed by fibrin matrix formation, which acts as a scaffold for cell infiltration. As a result of platelet degranulation, neutrophils are recruited to remove bacteria, foreign particles, and damaged tissues at the lesion region via chemotactic signals released by necrotic tissues and bacterial degradation products [12]. Approximately three days after the injury, the number of neutrophils decreases. Macrophages that coordinate all events evolved in response to damage, including resolving inflammation, host defense, removal of apoptotic cells, and cell proliferation tissue restoration [11].

The proliferative stage begins within two to ten days after injury and involves migration and interaction between different cell types. The process includes angiogenesis, granulation tissue formation, and migration of keratinocytes to the lesion region known as epithelialization [11]. The granulation tissue composed of fibroblasts, myofibroblasts, and endothelial cells is responsible for the synthesis of fibrillar components, wound contraction, and the neo-angiogenesis process [13]. Fibroblasts also form collagen by synthesizing and depositing ECM proteins that eventually contribute to scar formation. Any disruption in this process can cause abnormal scarring; hence, it is pivotal to maintain a balance between ECM protein deposition and degradation [2].

Remodeling, the last phase of healing, begins about two to three weeks after an injury and can last for over a year. During this phase, all previous stages are inactivated, focusing on the extension of new epithelium and apoptosis of unnecessary blood vessels, fibroblasts, and inflammatory cells, leaving a region rich in collagen and other ECM proteins, resulting in scar maturation [11]. Gradually, within 6 to 12 months, type III collagen in ECM is replaced by type I collagen, and effective interaction between the dermis and epidermis combined with additional feedback continues to regulate skin integrity and homeostasis [14].

The healing process is usually related to and determined combinedly by the nature of pathological processes and degree and status of host and environment [15]. Healing of the wound can also be affected by systematic factors such as the age of the patient, the presence and absence of vascular, metabolic, and autoimmune diseases, and ongoing drug therapy [2]. Time is also another significant parameter in wound healing and repair. The ability to heal gradually diminishes with age for various reasons, including decreased skin strength and elasticity and significantly reduced blood flow because of a sedentary lifestyle and smoking [15]. Moreover, in many cases, patients with HIV, cancer, and malnutrition all have a degree of immunosuppression that can lead to delayed wound healing [16]. Apart from that, the use of drugs that can impair the inflammatory response can impede the healing cascade. Hence, to mitigate these constraints, regenerative medicine through tissue engineering technology has emerged with several opportunities to accelerate and promote wound healing and regeneration [15].

## 3. Skin Tissue Engineering and Regenerative Medicine

Tissue engineering, an emerging interdisciplinary field in biomedical engineering, aims to regenerate new biological materials for replacing diseased or damaged tissues or organs [17]. A source of cells, biomaterials, and biomolecules is required along with an artificial extracellular matrix upon which cells can be supported and enriched for further survival. The engineered skin substitutes can be classified into three categories (i) based on materials: Biological, synthetic, and biosynthetic; ^3^ based on covering time: Temporary and permanent; and (ii) based on layer: Epidermal, dermal, and bilayered skin substitutes, and can be used in combating acute and chronic skin wounds [18]. The first attempt in skin tissue engineering was taken by fabricating a cultured epidermal autograft (CEA) from the small piece of skin containing cultured human keratinocytes, which were later used clinically for burn treatment. However, these autologous cultured sheets exhibited certain demerits such as graft instability, prolonged culture time, formation of fragile skin after healing, and lack of dermal matrix support, limiting its application [2]. Since then, several innovative approaches have been adopted to create skin substitutes with essential properties including being easy to handle and apply to the wound site; enabling vital barrier function with appropriate water flux; being sterile, non-toxic, and non-antigenic; and evoking minimal inflammatory reactivity [4]. Besides, they should be incorporated into the host with minimal scarring or pain and facilitate angiogenesis while still being cost-effective [19]. Thus, a number of approaches based on the choices of cell types (keratinocytes, fibroblast, stem cells), their source (allogenic or autologous), and choice of biomaterials for matrix formation (synthetic, natural, ECM based) have been made to improve skin substitutes [4].

Other than tissue engineering, regenerative medicine has grown out of diverse disciplines such as surgery, organ transplantation, biomaterial science, developmental biology, and stem cell biology [20]. At present, the scope of regenerative medicine in wound treatment includes technologies and approaches that induce the body to redevelop missing tissue, regardless of their conformation and engineered tissue or organs designed fully to replace the missing structures [17]. The merging of tissue engineering and regenerative medicine occurred with stem cell and therapeutic cloning research. This merging has been abetted by recognizing that various engineered skin constructs, some of which were originally designed to engraft and serve as replacement structures, stimulate endogenous processes that remodel the construct with the body’s tissue [20]. Another scope of regenerative medicine is to incorporate the relationship between tissues, organs, and systems, even the body as a whole, that enables us to combine several different molecular approaches into one course of treatment. The example includes reducing inflammation, stimulating tissue development pathways, recruiting endogenous stem cells, modulating immune function, and stimulating new blood vessel formation [4]. Hence, a deeper understanding of TERM, including the relationships between systems, will connect clinicians with scientific engineering skills to commercial teams and guide new technologies towards safe and effective treatment strategies in wound healing [20].

## 4. Techniques of Skin Tissue Engineering

In skin tissue engineering, new techniques such as bioprinting, bio-fabrication, and bio-inking coupled with advances in DNA microarray, proteomics, and stem cells have allowed the generation of skin replacements exploration [11]. The main elements of tissue engineering include biomaterials, cells, growth factors, other signalling molecules, and engineering components such as scaffolds, pumps, tubes, and bioreactors [2]. At present, 3-dimensional (3D) scaffold constructs made via bio-fabrication techniques exploit the field of skin tissue engineering as a key component in the wound healing process. Scaffolds play a unique role in the repair and restoration of disintegrated tissue by providing a suitable platform for various factors associated with cell survival, proliferation, and differentiation [9]. It can be constructed from natural and/or synthetic biomaterials, either materials that remain stable in a biological environment or materials that degrade in the human body [2]. Several techniques have been adopted for their constructions, but the four main approaches that are widely used include: (i) Sheets of cells secreting ECM [21]; pre-made porous scaffolds of synthetic, natural, and biodegradable biomaterial; (ii) decellularized ECM scaffolds, and (iii) cells entrapped in hydrogels [9]. The main objective of the scaffold is to represent the matrix as similar as possible to the native ECM as all cells are in close contact with ECM, which provides structural support to cells and tissues, stimulating migration proliferation, apoptosis, survival, and differentiation [2]. Hence, based on this, different parameters such as physio-chemical properties of the pristine materials, mechanical properties, shape, structure, pore sizes, and distribution need to be considered while fabricating scaffolds for wound healing and regeneration [22].

In practice, the techniques of fabrication of 3D scaffolds are subdivided into conventional and rapid prototyping (RP) methods (Table 1), each producing different scaffolds with different characteristics [23]. Scaffold fabrication using conventional techniques include the construction of porous polymeric structures such as substrates for cell adhesion; however, it is challenging to obtain complex structures of microscale (containing an environment suitable for cell survival and function) and macroscale (permit the coordination of multicellular process, provide adequate transport of nutrients, and possess mechanical properties) using conventional methods [24]. On the other hand, the RP scaffold fabrication technique provides versatile opportunities for skin tissue engineering. It allows the independent control of macroscale and microscale features, facilitating the fabrication of multicellular structures needed for complex tissue functions [24]. Moreover, 3D vascular bed fabrication is possible using the RP technique, which allows the support of massive tissue formation. In addition, RP provides an opportunity to combine fabrication technique with clinical imaging data, increasing the possibility of producing a bulk number of customized scaffolds in designated designs [25].

Apart from the conventional fabrication technique, 3D bioprinting involving the use of computer-controlled deposition of cells into precise 3D geometrical patterns has shown promising accuracy in cell delivery to replicate natural skin anisotropy [35]. Tarassoli et al. (2018) describe two main approaches to arranging cells in 3D patterns. The former is a top-down approach whereby cells are co-arranged with biomimetic scaffolds with tissue maturation in a bioreactor [32]. On the other hand, the later involves a bottom-up fabrication technique in which a temporary support instigates secretion of the matrix by cells themselves. Despite the chosen 3D bioprinting technique, the functionality of a successfully engineered skin largely depends on the biomaterial and cells used. Some aspects that are considered during biomaterial selection include biocompatibility, biodegradability, bio inertness, strength, durability, and ductility [37,38]. For bioprinting purposes, biomaterials should be ‘printable’ depending on their rheology (divided into aspects such as shear thinning and viscosity) and cross-linking abilities (through a chemical, physical, stereo complex, or ionic mechanism) [35]. Cell selection is the second key component of bioprinting. So far, much of the research has focused on using keratinocytes, which can be propagated easily in cell culture and fibroblasts, which have multilineage potential [39]. Besides, stem cells have been sought out as potential alternatives as they can both self-renew and differentiate into multiple cell types [35]. Although bioprinting technology offers promising outcomes in skin tissue engineering, a lack of understanding of bioink compatibility and biomaterial characteristics can be a major limitation in fully realizing this technology [40]. Hence, it is critical to innovate properties of bioinks that facilitate easy bioprinting processes, while preserving the viability and function of the printed tissue constructs

## 5. Components of Skin Tissue Engineering and Regenerative Medicine

**Figure 2:**
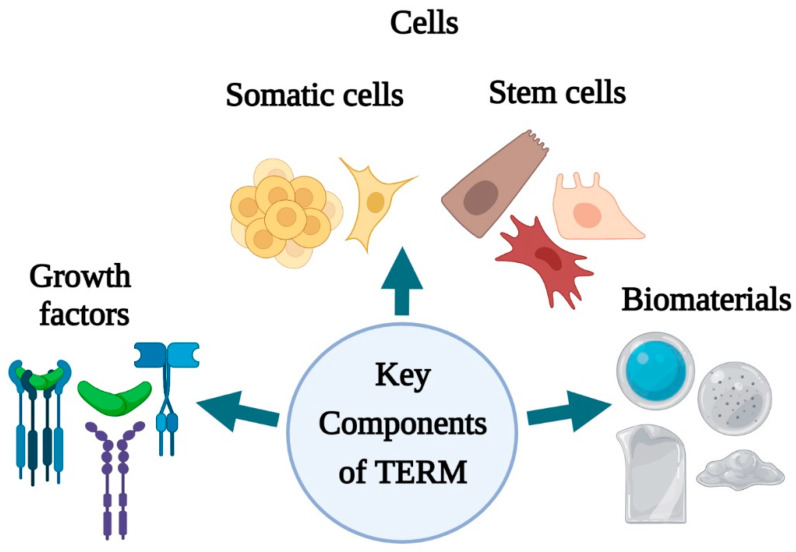
Key components of skin tissue engineering and regenerative medicine (created with BioRender.com).

Tissue engineering and regenerative medicine (TERM) can be considered a multidisciplinary and emerging field in technology used to regenerate damaged organs, produce complex tissues, and maintain normal cell homeostasis [17]. TERM aims to design new tissues and organ replacements that closely mimic a typical physiological environment for cells. It has caused a revolution in the present and future therapeutic possibilities for acute and chronic wound healing, improving restoration of biological function and rehabilitation [20]. Advanced multidisciplinary TERM approaches involving growth factors, stem cells, and biomaterials are being adopted to induce regeneration or indirectly change the wound environment and stimulate healing [2]. The key components of regenerative medicine and tissue engineering (growth factors, cells, stem cells, biomaterials) (Figure 2) have unveiled several perspectives for skin tissue engineering and regeneration that can be used to address different stages of wound healing.

### 5.1. Growth Factors

Growth factors (GFs) are defined as the biologically active polypeptides that control tissue repair via interacting with specific cell surface receptors. They play a prominent role in cell migration into the wound area, promote epithelialization, initiate angiogenesis, and stimulate matrix formation followed by remodeling the wounded area [11]. Epidermal growth factor (EGF) [41], basic fibroblast growth factor (bFGF) [39], transforming growth factor-beta (TGFβ_3_) [40], platelet-derived growth factor (PDGF) [42], and vascular endothelial growth factor (VEGF) [43] are some of the GFs that contribute to the wound-healing process. EGF secreted by platelets, macrophages, and fibroblasts plays an important role in epithelialization and stimulates growth of keratinocytes [44]. In an in vivo study conducted by Jeong et al. (2020) on healing diabetic mice wounds, EGF encapsulation in gelatine-alginate coacervates showed improved wound healing capacity via enhanced granulated tissue formation and cell migration and re-epithelialization [41]. According to Li et al. (2018) and Xu et al. (2020), genetically modified TGFβ_3_ and PDGF combined with synthetic biomaterial both accelerated in vivo wound repair in a rabbit and mice model, respectively, but through specific mechanisms. TGFβ_3_ contributed to reduced scar formation by decreasing the proliferation of myofibroblast and the ratio of type I to type III collagen [40,42].

In contrast, the role of PDGF in wound healing was to work as a chemoattractant for macrophages and fibroblasts. It also stimulates them to express growth factors, including TGFβ, which is essential in inflammation, granulation tissue formation, epithelialization, matrix formation, and remodelling [42]. Besides, bFGF belonging to the FGF protein family is widely studied and has confirmed its role in the proliferation of both epithelial and mesenchymal cells and promoted angiogenesis [39] and collagen deposition in the treatment of diabetic mouse wound model [45]. In addition, a VEGF-loaded hydrogel reported an improved chronic wound healing process of an infected full-thickness skin model via the promotion of angiogenesis, collagen deposition, macrophage polarization, and granulation tissue formation [43]. Therefore, several researchers have proven the role of each growth factor in wound healing; moreover, a handful of studies verified the potential of using growth factors in combination with carriers for effective delivery in maximizing wound healing. Table 2 depicts the function of various GFs secreting from different sources and their outcome in wound healing. Although in vivo applications of growth factors have been shown to accelerate wound healing (Table 2), therapeutics have several drawbacks. Its application has been limited due to its short life span [46], the possibility of bursting inside the host body, and the high price of recombinant GFs [47]. Hence, further studies on the interplay between cells and GFs are encouraged to achieve effective combination treatment in the wound healing cascade considering its dynamic nature.

### 5.2. Cells and Cellular Skin Substitutes

In the tissue engineering triangle, both cells and cellular skin substitutes (both differentiated and stem cells) have exhibited great potential by providing all the elements required for skin regeneration such as cells, mediators, and materials mimicking ECM [3]. The use of viable cells cultured in special conditions are used to produce cell sheet substitutes that contribute to wound repair. Among the available differentiated human cells, fibroblasts and keratinocytes are the primary sources for epidermal and dermal substitute production, respectively [49]. Fibroblasts are spindle-shaped cells and are widely distributed in most types of tissue, particularly connective tissue. These cells are of mesenchymal origin and function to regulate ECM turn over under normal condition [50]. In injured tissues, fibroblasts are activated and differentiated into myofibroblasts that contract and participate in critical wound healing by synthesizing most ECM structural components such as collagen, elastin, laminin, and glycosaminoglycan [50]. Myofibroblasts are an intermediate cell type between fibroblasts and smooth muscle cells (SMCs). These cells with ultra-structure appear in the early phase of granulation tissue formation, become most abundant in the proliferation phase of wound healing, and progressively disappear in the later stage of recovery by apoptotic mechanism [51]. Hence, fibroblasts [50] and myofibroblasts [51] are critical in supporting key healing processes like breaking down the fibrin clot and creating new ECM and collagen structures to support the other cells associated with effective wound healing and contracting the wound. As for keratinocytes, they are major cellular components of the epidermis, and their phenotype varies depending on the stage of the maturation process. They are responsible for recruiting, stimulating, and coordinating the function of multiple cell types involved in healing, recapitulating the epidermal barrier layer of skin, and representing an effective defense barrier against the external environment [52]. In response to the disruption of the barrier, keratinocytes release prestored interleukin 1 (IL-1), a group of 11 cytokines that acts as both an autocrine and paracrine signal that activates and increases keratinocyte migration and proliferation, as well as mobilises surrounding cells to aid in healing [52].

**Figure 3:**
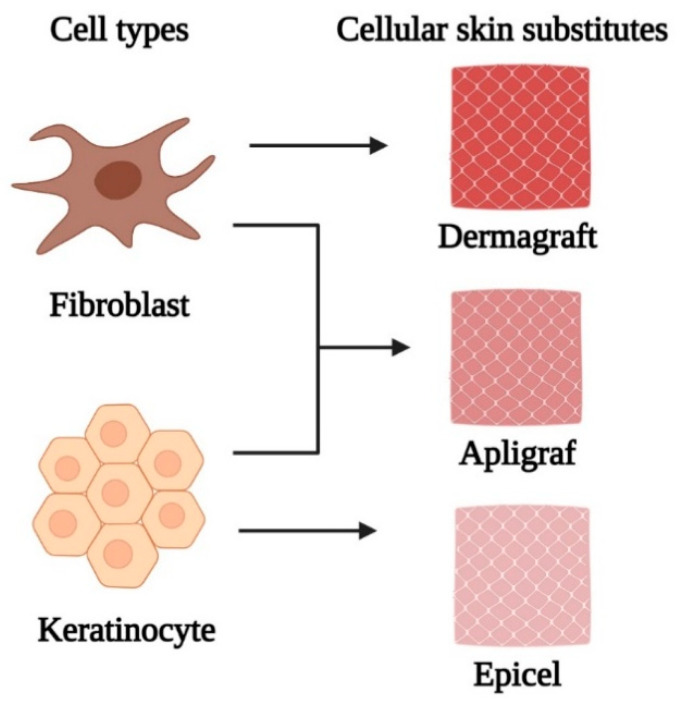
Cellular skin substitues made from fibroblast and keratinocyte (created with BioRender.com).

Taking advantage of the wound healing properties of fibroblasts and keratinocytes, specific cell composition constructs have been developed according to the treatment target for dermal and epidermal regeneration [2]. When applied to the wounded area, the cells supply signalling molecules, growth factors, and ECM proteins that aid healing [52]. Through a paracrine crosstalk mechanism, fibroblasts and keratinocytes communicate with each other, leading to to cell recruitments and maintenance of skin homeostasis, which is desirable for complete wound healing. For this purpose, several double-layer dermal cellular skin substitutes have been synthesised commercially, incorporating both fibroblasts and keratinocytes for the repair and regeneration of chronic wounds [53]. EpiCel, Dermagraft, and Apligraf (Figure 3) are some of the instances of commercially available cellular skin substitute products that incorporate both keratinocytes and fibroblasts, respectively. These novel products are assembled according to their specific conformation and structure; in particular, pore sizes and their distribution are essential in providing a suitable matrix for effective cell migration and arrangement [54]. The novelty of these products represents a basis for revascularization, forming a proper microenvironment for cellular migration and proliferation [55]. For instance, Apligraf is the first bilayered living cellular skin substitute construct approved by the US Food and Drug Administration (FDA) containing keratinocytes and fibroblasts derived from donated human neonatal foreskin in bovine type 1 collagen matrix. This grafted skin substitute product is used to promote the healing of ulcers that have failed standard wound care and has shown improved results in randomized, controlled clinical trials in the reduction of healing time and increasd the incidence of complete wound closure in treating both diabetic foot ulcers and venous leg ulcers [54]. Although these products provide effective cell types required for healing, a matrix for non-healing wounds, cytokines, and growth factors similarly to healthy human skin, it is not applicable to all chronic injuries. This is because special wound bed conditions are necessary to achieve optimal effectiveness in wound repair. In addition, the use of autologous skin cells for wound healing is restricted due to limited donor sites [2]. To overcome this limitation, stem cells (SCs) have emerged as a possible alternative in repairing injured tissue as they have self-renewal capacity, multilineage differential potential, and can be retrieved from several tissues such as embryonic, fetal, and adult tissues.

### 5.3. Stem Cells

The use of stem cell (SC) has become a promising new approach in tissue engineering and regenerative medicine for skin injury treatment. Stem cells can be defined and characterised based on their capacity for self-renewal, asymmetric replication, and potency [56]. They are attributed with the ability to replenish lost cells throughout the lifespan of an organism via unlimited replication, providing a population of sister stem cells [57]. The main clinical focus of stem cell application in wound healing is to accelerate the healing process, prevent wound contracture and scar formation, and initiate earlier wound closure and regeneration of skin and its appendages. Besides, stem cells can secrete pro-regenerative cytokines, making them an attractive agent for treating chronic wounds [56]. Among the primary sources of cells that are in use for wound healing and the regeneration of injured skin are embryonic stem cells (ESCs), adult stem cells, and induced pluripotent stem cells (iPSCs) [58].

ESCs are pluripotent stem cells obtained from mice, human, and non-human primates and derived from the inner cell mass of the pre-implantation blastocyst (35-day-old embryo) [59]. They can differentiate into various cell types, including neural cells, blood cells, adipocytes, chondrocytes, muscle cells, and skin cells [11]. ESCs and their progenitors in the epidermal basal layer and terminal hair follicles make them an attractive source in wound therapies. Besides, ESCs and their progenitors are also considered autologous cell sources for chronic wound healing [60]. Furthermore, in the presence of a selected medium containing growth factors, ESCs can be differentiated into keratinocytes. Later these keratinocytes form multi-layered epidermis in culture, making them a potential cell type for bioengineered skin [60]. In addition, Chen et al. (2019) reported that exosomes derived from human ESCs accelerated the pressure ulcer wound healing process and promoted local angiogenesis at the wound site in aged mice by rejuvenating endothelial senescence [61]. The study further states that exosomes derived from human ESCs can behave like their parental ESCs and contribute to antiaging and regenerative medicine via transfer and encapsulation of bioactive molecules to target cells. Although in vivo study of ESCs shows promising results in wound healing, its clinical use is limited by the ethical controversy revolving their procurement process as it can induce damage to the human embryo [62]. Instead, much interest is being developed towards ASCs and iPSCs as they possess minimal ethical concerns relating to procurement and clinical translation. Adult stem cells are most widely used in wound healing and skin regeneration owing to their remarkable proliferative capacity, long-term self-renewal potential, and ability to differentiate into other lineages. Skin, heart, liver, and bone marrow serve as potential sources of adult stem cells [56]. Among different types of stem cells, mesenchymal stem cells (MSCs) and adipose-derived stem cells (ASCs) have gained considerable attention as suitable candidates to improve tissue regeneration [60]. MSCs harvested from various sites (bone marrow, adipose tissue, amniotic fluid, and dermis) are involved in all three phases of wound healing and improve the process of healing by immune modulation, the production of growth factors (vascular endothelial growth factor, hepatocyte growth factor, fibroblast growth factor), enhancing neovascularization and re-epithelialization, stimulating angiogenesis, and accelerating wound closure [63].

A study by Ferreira et al. (2017) reported that adipose tissue-derived MSC secrete extracellular vesicles that accelerate migration and proliferation of fibroblast and keratinocyte, promoting wound healing in vivo [64]. Moreover, Li et al. (2017) demonstrated that activated MSCs promote acute incision wound repair in a mouse model, as reflected in regained tensile strength [65]. Compared to MSCs, ASCs are more efficient due to their high accessibility with minimal invasiveness and minimal ethical limitations [66]. A recent study by Yu et al. (2018) highlighted that cell sheets composed of ASC transplanted in a nude mice model demonstrated an improvement in wound healing and reduced scar formation [67]. Moreover, the study reported that applying ASCs in the cell sheet format increased cellular survival within wound tissue during the healing process. In addition, the transplanted ASCs were eliminated by 28 days after wounding, thus minimizing the long-term side effects of cellular transplantation. Apart from MSCs and ASCs, bone marrow-derived stem cells (BM-SCs), and human umbilical cord-derived mesenchymal stem cells (hUC-MSCs) are good candidates for the treatment of different types of wounds [11]. hUC-MSC isolated from umbilical cord lining tissue represents another mesenchymal stem cell population [62]. Notably, the epithelial cells belonging to the umbilical cord possess stem cell-like properties and can differentiate into any form of stratified epithelium required for improved wound healing [56]. Moreover, hUC-MSC contains characteristic cell surface markers (CD105, CD73, and CD 90) that facilitate growth factor secretion for wound healing [68]. In vitro experiment showed that the treatment of diabetic wounds with hUC-MSC showed higher cell proliferation and collagen synthesis than fibroblasts [69]. Furthermore, clinical application of hUC-MSCs employed in treating chronic diabetic ulcers significantly decreased both ulcer size and time required for wound healing to occur [68]. After seeding on an acellular amniotic membrane scaffold, hUC-MSCs promoted tissue regeneration and improved wound healing outcomes. The scaffolds conferred anti-adhesive, bacteriostatic, and epithelialization properties, and attenuated the wound pain reported by patients [68]. BMSCs are a group of skeletal progenitor cells originating from bone marrow and can differentiate into many cell types such as adipocyte, osteoblast, and chondrocyte [70]. In vitro study on BMSC use reported cell proliferation and acceleration in human epidermal keratinocytes [71]. Moreover, the same study reported that subcutaneous injection of BMSC into a full-thickness wound significantly improved epidermal thickening in mice with a prominent keratinized layer, accelerating re-epithelialization and significantly improving healing quality. Although stem cells can treat various skin injuries, there are still concerns surrounding vital consequences. Table 3 demonstrates the use of different stem cells in wound therapeutics.

Stem cells can differentiate into many cell types, which makes them considerably promising. However, this ability may also lead to tumor formation [2]. Besides, differentiation of stem cells into the wrong kind of tissue is another concern that requires careful consideration regarding therapeutic stem cells use [11]. In regenerative medicine, iPSCs emerged as the newest class of pluripotent stem cell that combines the advantages of ESCs and MSCs [2]. The iPSCs are like ESCs in terms of their morphology, self-renewal capacity, and differentiation. They can differentiate between all types of cells from the skin to nerve and muscle. Taking advantage of this characteristic, potential progress has been made in the differentiation of iPSCs into skin cells, including melanocytes, fibroblasts, and keratinocytes, to engineer skin substitutes [56]. Intradermal injection of human iPSC-derived endothelial cells in a murine excisional wound healing model was reported to promote angiogenesis, accelerate wound closure, and increase wound perfusion [72]. Besides, a study by Wang et al. (2019) highlighted that NANOG and LIN28 transcription factors synergize to improve reprogramming latency at least by a week and act as main drivers of reprogramming in cell epithelialization [73]. 

Moreover, extracellular vesicles derived from human iPSC-MSC tested in mice via intravenous injection (IV) exhibited accelerated wound healing as measured by epithelialization after IV delivery of 1 × 10^6^ cells [21]. Although iPSCs have shown promising performance to accelerate wound healing in rodent models, further exploration is essential to study their development and safety profile, particularly in tumor formation. This is because reprogramming adult somatic cells and inducing subsequent differentiation in the desired cells can be complex as the process involves altering genomic stability and differencing capability [74]. Besides, these alterations can often result in a heterogeneous cell population with the undifferentiated ability and their in vivo self-renewal can give rise to teratoma [2]. Hence, a better understanding of these cells is required to allow clinical translations for the safety of patients.

### 5.4. Biomaterials

In tissue engineering, biomaterials play a prominent role in unlocking the regenerative potential innate to human tissues/organ, restoring deteriorated state, and re-establishing normal bodily function [76]. Biomaterial science and engineering have witnessed tremendous growth in the past five decades due to vast investment in developing new products [77]. In a broader sense, biomaterials can be defined as material devices or implants used to repair or replace native body tissues or as a provisional scaffolding material adopted to construct human-made tissues or organs [76]. Using biomaterial in tissue engineering aims to provide temporary mechanical support and mass transport to encourage cell adhesion, proliferation, migration, and differentiation and control the size and shape of the regenerated tissue [78]. Moreover, biomaterials known as temporary scaffolds act as an ECM template that is actively involved in delivering cues to the cells that form the regenerated tissues [4]. Numerous approaches are adopted for designing matrices, comprised of innovative biomaterials possessing two crucial traits: Biocompatibility (the materials must hold minimal toxicity and immunogenicity) and biodegradability (the materials must be easily removable upon completion of their function) [78]. Furthermore, they must also possess the ability to interact with a biological environment and particularly modulate cellular response [4]. Hence, biomaterials have become an active part of cellular function regulation and act as a support for tissue regeneration or a platform for drug delivery. There are various biomaterials available from living (animals/humans), vegetal, and synthetic sources; nevertheless, therapeutic biomaterials can be commonly classified into two categories: Natural and synthetic biomaterials [77]. Table 4 summarizes the examples of two types of biomaterials used according to their advantages and disadvantages in skin tissue engineering.

Natural biomaterials derived from living and vegetal sources are widely studied in tissue engineering due to their bioactivity, biocompatibility, tunable degradation, and structural resemblance to native ECM tissue [76]. Their application within biological systems releases low cytotoxic products upon degradation and promotes biological recognition to support cell adhesion and function [77]. Collagen, gelatin, elastin, fibrin, cellulose, and chitosan are examples of abundantly used natural biomaterials derived from protein sources and polysaccharide sources [9]. Collagen, the most abundant protein in the body and the major component of ECM, has wide application in wound healing and skin regeneration. This is due to its good mechanical properties and biocompatibility; however, collagen is susceptible to crosslinking and sterilization procedures [78]. Besides, it degrades rapidly upon treatment with collagenase, gelatinase, or other proteins [78]. Collagen hydrogels, microfiber collagen scaffolds, and electrospun collagen nanofibrous scaffolds are just a few examples of formulations for wound healing and skin regeneration [9]. Collagen has also been exploited to produce nanofibrous scaffolds via the electrospinning technique as skin substitutes for collagen type I and type III or to coat scaffolds made from other materials like gold nanoparticles and increase their biocompatibility [79]. Moreover, clinical studies of collagen dressing loaded with antibacterial components have reported improved healing rates in patients suffering from diabetic foot ulcers [80]. Gelatin is a partially hydrolyzed version of collagen and retains most collagen chemical functionality [78]. It is composed of triple amino acids of glycine proline and hydroxyproline and has been added to various biomaterials to enhance cell scaffold interactions through its Arginine-Glycine-Aspartic acid sequences, which are easily recognized by integrin receptors of cell membranes [81]. Gelatin nanofibers, along with several scaffolds like gelatin alginate sponges, gelatin-containing EGF, and gelatin films, showed possible applications in the treatment of burnt skin healing and regeneration [78]. Moreover, clinical study of a collagen dressing loaded with antibacterial components has reported an improved healing rate in patients suffering from diabetic foot ulcers [80]. Furthermore, gelatin exhibits lower antigenicity than collagen, a suitable property for treating the wound with high infection risk. In addition, it is cheaper than collagen and has higher solubility in most solvents [82]. Despite having potential wound healing properties, both collagen and gelatin lack high mechanical resistance; hence, they cannot be used as scaffolds for hard tissues [2].

Elastin and fibrin are widely used biomaterials for designing scaffolds (in both mono and bilayered form) for skin substitutes [102]. The skin constitutes 2 to 4% elastin, which is responsible for the elasticity of skin tissue [103]. The formation of elastin is prolonged (4 to 5 years) in injured tissue, which compromises its distribution and morphology in wound healing, resulting in a lack of native function [102]. Besides, most of the collagen-made skin substitutes available in the market lack elastin; therefore, they suffer from low elasticity, high contraction during wound healing, and scar tissue formation [78]. Recently, elastin blending with collagen has demonstrated biological compatibility, tissue integration, and earlier neovascularization in mice compared to the commercially available Integra Dermal Regeneration Template (manufactured by Integra company in U.S.), while also promoting fibroblast and keratinocyte proliferation in vitro [104]. Fibrin, another promising biomaterial obtained from fibrinogen, can be used as a temporary scaffold due to its immunocompatible nature. Fibrin gels can facilitate the secretion of growth factors like cytokines or other bioactive molecules that control the process of adhesion, proliferation, cell migration, differentiation, and ECM production in the wound healing process [78]. Moreover, silk fibroin, the main structural protein of silk, can be used in tissue engineering applications due to its semi-crystalline structure and high mechanical strength [92]. Despite the lightweight nature of silk, its tensile strength is superior to those of other biopolymers such as collagen; therefore, it can be easily spun into nano fiber scaffolds that support human fibroblasts and keratinocytes cell adhesion and spreading in plate morphology [83]. However, there is a dilemma regarding the biocompatibility of silk fibroin, and sericin found in silk could induce allergic reactions, immunogenicity, and the release of some tumor necrosis factor [92].

Among polysaccharide-derived biomaterials, cellulose and chitosan are widely used in wound healing and regeneration. Alginate is a naturally occurring biopolysaccharide obtained from seaweeds. They are well known and widely used for wound dressing and management systems due to their biocompatibility, gelling, and swelling nature, which exhibits a moist microenvironment at the wound site, favoring the proper healing procedure as well as reducing the healing time [84]. This unique characteristic of alginate allows the formulation of wound dressings like hydrogel, films, foams, gels, and nanofibers, making it a useful biopolymer of potential importance that can triumph over the shortcomings associated with other biopolymers used in skin tissue engineering application [83]. Cellulose is the most abundant natural biopolymer found in plants and certain microorganisms like *Acetobacter xylinum*. Its chemical uniqueness, the shape of flexibility, ease of processing, mechanical strength, and biodegradability makes it a potential scaffold material for skin injury treatment [104]. Cellulose/collagen hybridized dressing is reported to have benefits compared to a conventional wound dressing in diabetic foot ulcer treatment as it contributes to improved re-epithelialization of injured tissue [105]. Chitosan derived from chitin is the second-most abundant biomaterial after cellulose that can be used alone or in combination with other materials like collagen, fibrin, and gelatin in the form of sponges and hydrogels [87]. It is widely used for wound dressing due to its antibacterial, antifungal, mucoadhesive, and analgesic properties that do not trigger inflammation or infection [78]. Despite having promising potential in wound healing and regeneration, naturally derived biomaterials can possess weak mechanical strength and inconsistency in composition and properties associated with batch production due to their origin in living beings [2]. Besides, most natural biomaterials are susceptible to degradation, particularly after extraction and exposure to light and heat [77]. In addition, natural biomaterials derived from protein sources provide excellent growth media for microbes, and their sterilization may have adverse effects on the structure and properties of the material [76]. To overcome these limitations, recent advances in tissue engineering and fabrication have led to the development and use of synthetic biomaterials to mimic ECM systems of living origins.

Synthetic biomaterials used in tissue engineering are produced in labs from hydrocarbon building blocks [77]. Although they lack the inherent cell interaction moieties present in natural biomaterials, they offer desirable options for controlling shape, architecture, and chemistry to generate alternatives to or mimic ECM systems [78]. Besides, their ability to be reproduced industrially on a large scale by controlling parameters such as molecular weight and degradation time, and their modification of chemical properties to produce derivatives with improved adhesion, cross linking, and biodegradability has made them interesting for skin regeneration applications [77]. Synthetic polymers such as polycaprolactone (PCL), polylactic acid (PLA), polyglycolic acid (PGA), polyethylene glycol, and related copolymers like polylactic-co-glycolic acids (PLGA) are used to compose matrices individually or as composites for tissue regeneration due to their greater compatibility with body tissues [77].

PLA is a U.S. Food and Drug Administration (FDA)-approved biodegradable polyester derived from rice and corn [78]. Despite having limitations like poor cell interaction, slow degradation rate, low elongation, and hydrophobicity that can initiate an inflammatory response, PLA has been extensively employed for tissue engineering scaffold application to deliver cells to the wound site [100]. PCL is another popular biomaterial used in numerous studies for skin tissue engineering with a slower degradation rate compared to PLA and PLGA [78]. Initially, PCL was used to produce degradable films and molds; however, at present, with the advent of electrospinning technology, it is used to create absorbable sutures, drug delivery systems, and scaffolds for tissue regeneration [93]. Considering its slow degradation time and acidic degradation products, PCL can be regarded as a potential option to modify natural biomaterials with slower degradation and improved mechanical properties [94]. PGA is a crystalline polymer that is not soluble in many organic solvents [78]. Its hydrophilic nature contributes to rapid mechanical strength loss and allows reabsorption four weeks after implantation [9]. Moreover, PEG as a synthetic biomaterial is desirable for its structural and compositional property; however, it lacks interactive cell character [78]. Such a bioinert nature of PEG as a blank template can be modified using different moieties to ensure the additional requirements of a skin structure-like cell adhesion, short-term degradation and minimum inflammation are met [9]. Furthermore, poly (3-hydroxyalkanoate)s (PHAs) are naturally occurring aliphatic polyesters found in many living organisms in the form of intracellular granules for storing carbon and energy when they are subjected to stress conditions or lack of nutrient [106]. Currently, there is a growing interest in the use of PHA for reconstructive surgery and tissue engineering applications due to their renewability, biocompatibility, non-toxic degradation, and biodegradability [107]. Apart from the mentioned biomaterials, composite systems containing a unique combination of natural and synthetic biomaterials with bioactive compounds are being fabricated and studied for improving cell growth and healing effectiveness. Bioactive glasses such as poly 3-hydroxyoctanoate (45S5 Bioglass) and mesoporous bioactive glass (MBG) blended with PLGA copolymer demonstrated potential evidence in in vitro and in vivo scaffold neovascularization. Combining both provided a suitable microenvironment for cell growth and accelerated blood clot time by controlling anti-inflammatory agents [9].

Following any injury, common cellular and molecular events exist for tissue repair; however, each tissue commonly presents a unique cascade of the wound healing process [76]. Most tissue healing phases involve multiple signaling components that coax the cells under spatial and temporal control leading to optimum tissue regeneration [4]. Hence, a better understanding of the characteristics and design of biomaterials is essential to fabricate matrices for skin injury treatment and regeneration.

## 6. Challenges and Limitations in Stem Cell Therapeutics for Wound Healing

As stem cell-based therapies hold the potential to enhance wound healing and regeneration, their pioneering scientific and medical advances always need to be carefully considered to make sure that they are both ethical and safe. At present, there are several concerns regarding stem cell therapeutics, which involve stem cell isolation and characterization, understanding stem cell mechanism, its culture condition, mode of delivery, efficacy, and immunological rejection [108]. First, the concern of the utmost importance is in regard to stem cell propensity towards self-renewal and differentiation, which is highly influenced by their local environment. However, it is difficult to interpret how a cell population of cell culture expands or may behave in animal models and clinical studies [109]. Besides, stem cell isolation and characterization are critical due to their low survival rates, and the aseptic culture of stem cells requires highly experienced personnel and sophisticated laboratory techniques [109]. Contaminated stem cells may result in complications, particularly in the case of immune-compromised patients. Immunological rejection is a significant barrier to successful stem cell therapeutics as the immune system might recognize the transplanted cells as foreign bodies, triggering immune reactions resulting in transplant rejection [110]. In addition, depending on the appropriate application, stem cells require differentiation into the appropriate cell types before they can be clinically used, otherwise they can result in a harmful effect. As a result, stem cell-based therapeutics require regular monitoring of regenerated tissue throughout the patients’ complete recovery [109]. Moreover, under in vivo conditions, stem cells are exposed to hypoxic conditions. This change in oxygen level can induce oxidative stress, which might influence stem cell phenotype, proliferation rate, and pluripotency. Hence, it is vital to maintain in vitro culture conditions similar to their in vivo niches [108]. On top of that, Broder et al. (2017) mentioned that the treatment of stem cell therapy is relatively high; for instance, currently, a treatment may cost up to 40,000 US dollars for hematopoietic stem cell transplantation [110]. This is because products based on stem cells are manufactured at a low and individual scale following strict Good Manufacturing Practices (GMP). Costs usually account for all different necessary items, including multiple surgical procedures, maintenance of strict aseptic conditions, specific training of technical staff and maintenance of overall technical and staff support, specialized facilities, the need for producing small but highly unstable batches, and of course, the development of the different market strategies [111]. Therefore, it is a matter of concern as to whether these costs will be compatible with at least partial funding from governments, medical insurance companies, and public as well as private health private institutions with the current demographic of patients who require stem cell therapy.

Apart from the mentioned challenges, one major limitation of stem cell therapy in wound healing treatment is optimizing progenitor cell selection and delivery. Transplanting new and fully functional stem cell products requires the use of millions of working and biologically accurate cooperating cells and the environment [109]. It is observed that stem cells often suffer from decreased functionality, such as impaired differentiation capacity and alterations in the therapeutic gene expression and cytokine production. This is due to the pathological changes in the wound microenvironment because of systematic factors such as diabetes, vascular disease, and aging [4]. Hence, it is crucial to select the appropriate cell population to develop targeted therapies to restore the body’s natural regenerative potential. For instance, the effectiveness of unselected autologous ASC therapy in aged patients is compromised as the angiogenic potential of ASC is linked to reactive oxygen species (ROS) [56]. Oxidative stress caused by ROS can directly inhibit angiogenesis; therefore, a certain balance of oxidative stress is ideal and any disruption in this balance might reduce the ASCs capacity to modulate ROS levels within the wound, thereby reducing their therapeutic efficacy [56]. Thus, the selection of novel and appropriate cell sorting techniques that include a distinct surface marker signature for identification, the assessment of correlation between the transcriptional signature of the desired cells with the surface protein, and predicting the protein level association of gene expression profiles and surface markers can provide possibilities to enhance the functional ability of stem cells and will potentially make stem cell-based therapeutics available to a broader cohort of patients [112].

Once a favorable progenitor cell population has been identified, the challenge further lies with clinical translation in cell delivery. High cellular attrition and difficulties in tissue targeting have limited the approach of systematic cell delivery [113]. For wound treatment, local delivery has received increasing acceptance as the optimal delivery approach, and the therapeutic benefits of local MSC administration to treat wounds have been mentioned in pre-clinical and early clinical studies [113]. However, factors like oxidative stress, hypoxia, and inflammation make the wound microenvironment hostile to delivered cells. Besides, topical application via spray is limited by the delivery of nonprotected cells to the wound site and poor control of cell density and spacing [56]. An alternative delivery mechanism such as bioscaffold-based delivery (composed of natural and synthetic biomaterials) has been developed for stem cell transplantation to overcome these limitations and improve the therapeutic functionality of stem cells. It provides protection and controlled spatial cues for seeded stem cells to establish a functional niche and provide wound coverage.

## 7. Combinational Therapy Using Both Biomaterials and Stem Cell in Wound Healing and Regeneration Treatment

**Figure 4:**
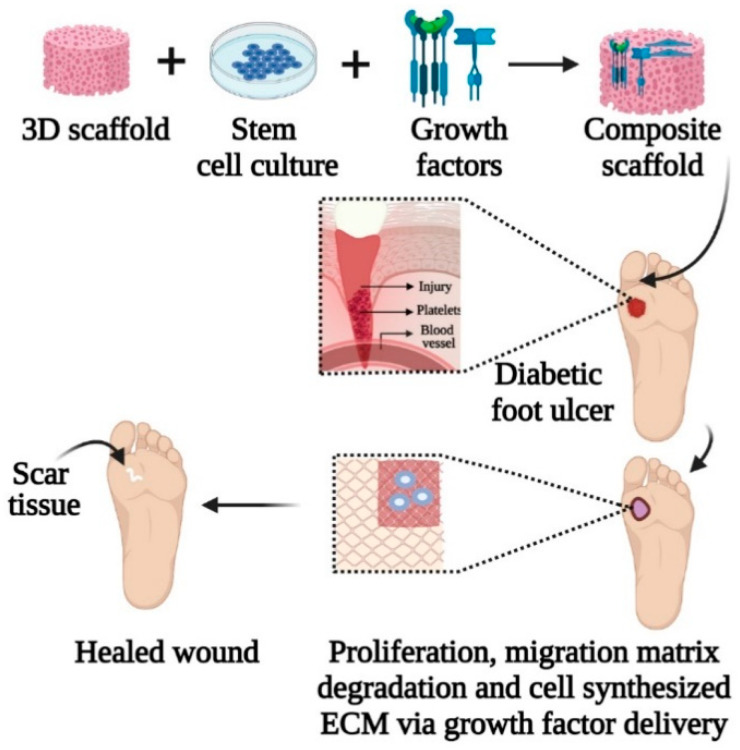
Biomaterial and growth factor incorporated stem cell therapy in diabetic foot ulcer treatment.

Biomaterials serve as a non-viable material in skin tissue engineering and regenerative medicine to repair malfunction tissues and organs. As mentioned earlier, stem cells require a specific environment for their survival and proliferation. Therefore, biomaterials have opened a new approach to regulating stem cells’ fate by mimicking the in vivo microenvironment via cell–matrix interactions. Scaffolds made from biomaterials can provide cell adhesion sites, maintaining the merits and characteristics of stem cells [58]. Compared to traditional 2D culture, the potential 3D biomaterial scaffold constructs provide a satisfactory microenvironment for stem cells by ensuring physical and chemical signals across the ECM [24]. Moreover, a well-designed scaffold with appropriate configuration can directly regulate cell signalling and initiate lineage-specific differentiation of stem cells by releasing chemical cues or the cell–matrix interaction [58]. Figure 4 illustrates biomaterial-incorporated stem cell therapy combined with growth factors in a diabetic foot ulcer injury for improved wound healing. The increasing demand for wound healing and tissue repair biomaterials is modified and exploited in combination with stem cells in various applications of wound healing treatment, as listed in Table 5.

In diabetic patients, a non-healing diabetic foot ulcer (DFU) can lead to leg amputation. Autologous stem cell therapy could solve this problem; however, stem cells under diabetic conditions are dysfunctional and restrictive in their wound healing capacity [122]. A study by Wen et al. in 2021 resolved this limitation by incorporating polyplex nanoparticles carrying the gene encoding for stromal-derived factor-1 alpha (SDF-1α) in collagen–chondroitin sulfate (coll–CS) scaffold, which enhanced the regenerative functionality of human diabetic adipose-derived stem cells (ADSC) [123]. After two weeks, it was observed that the gene-activated scaffold could restore the pro-angiogenic regenerative response in the human diabetic ADSCs similar to the healthy ADSCs on the gene-free scaffold. Diabetic ADSCs often show low expression of pro-angiogenic factors, which is considered a limiting factor in the application of cellular therapy [124]. In tissue engineering strategies, a construct’s ability to induce an enhanced angiogenic response is crucial for faster integration of the graft with the host environment [125]. Hence, the SDF-1α gene-activated scaffold can overcome the deficiencies associated with diabetic ADSCs, paving the way for autologous stem cell therapies combined with novel biomaterials to treat DFUs. Moreover, the transfected diabetic ADSCs also exhibited pro-wound healing features like active matrix remodelling of the provisional fibronectin matrix and basement membrane protein collagen IV [116], which is not only essential for supporting the adhesion and migration of cells, but also provides a scaffold for subsequent collagen deposition [126]. A similar in vivo study used human-induced pluripotent stem cell-derived smooth muscle cells (hiPSC-SMC) instead of ASC, combined with collagen scaffold to treat diabetic mouse [115]. The study concluded through an angiogenesis array that hiPSC-SMCs released a larger concentration of 28 out of 31 angiogenic cytokines such as VEGF-A, bFGF, and TGF-β1 compared to ADSCs. This phenomenon contributed to accelerated wound healing and improved wound architecture through cellular proliferation, increased macrophage production, and angiogenesis within 7 days of treatment. During wound healing, SMCs serve as a source of myofibroblast cells, which are essential in granulation tissue formation and are downregulated in chronic and diabetic wounds [127]. Besides, collagen scaffold increased survival of hiPSC-SMC in vivo and facilitated cell concentration in the wound center [128]. A total of 73% of the hiPSC-SMC in the wound center was Ki67 (a proliferative marker) positive compared with 2% in wound periphery, consistent with a proliferative SMC phenotype [115].

Apart from natural biomaterials, synthetic biomaterials such as PCL [119], PLGA [121], and PEG [120] have also been proven to be effective in wound healing when combined with stem cells. Such an instance is shown by Lopes et al. (2018) where bone marrow mesenchymal stem cells (BMSCs) were incorporated in a polycaprolactone (PCL)-based 3D scaffold to promote diabetic wound healing in mice [119]. Uniform cell distribution was observed throughout the entire scaffold, with cells displaying a radially aligned and porous pattern. When applied to the excisional wounded area, the BMSC-PCL scaffold showed more collagen deposition, new tissue formation, and newly formed blood vessels compared to the control group with the untreated wound by day 7. Complete re-epithelialization of the wounded area was also observed after surgery within 10 days, whereas there was still a large area of the dermis without epidermis coverage in the wounds of the control group. The advantage of BMSCs transplantation using the PCL scaffold includes acceleration of relative expression of growth factors and migration-related genes involved in wound healing, which contributes to angiogenesis, granulation tissue formation, and ECM secretion during wound recovery [119]. Hence, 3D scaffolds combined with stem cells can dramatically improve diabetic wound healing, minimizing the recovery timeline.

On the other hand, acute thermal injuries such as burns are still critical to managing as it comes with long-term hospitalization and expensive treatment options. To make burn injury treatment more accessible and efficient, promising strategies in tissue engineering using biomaterials (fibrin [117], gelatine [118]) and adipose stem cells (ASCs) have been widely used in clinical trials and laboratories as their contribution have been identified in the repair process of complex burn wounds including inflammation, granulation, and remodeling. A recent in vivo study by Zakrzewski et al. (2019) using enzyme crosslinked gelatine hydrogel with human adipose-derived stem cell (hASCs) spheroid reported improved wound repair in the murine burn model [118]. According to the study on day 14, the cell spheroid with hydrogel group showed the highest wound contraction rate of 55.3% compared to the control, which was 30.2%. The result demonstrates that the design concept of combining stem cells and gelatine hydrogel accelerated the wound healing process of the burn wound models. This prominently demonstrated that hASCs can differentiate into target cells involved in wound healing followed by secreting several growth factors such as PDGF, EGF, and TGF-β, which are involved in angiogenesis and gelatine scaffold, and provide the appropriate natural cell adhesion motif that supports cell migration as well as blood vessel infiltration making it a suitable topical regenerative biomaterial for wound repair [78,123]. Moreover, on day 10, the stem cell spheroid with gelatine hydrogel showed a lower discoloration rating and roughness scores attributed to the reduced extent of scab development promoting faster tissue regeneration [118]. Furthermore, live and dead staining result showed that gelatine enzyme crosslinked hydrogel has better biocompatibility with hASCs with an even distribution pattern and wide proliferation after 7 days of incubation without any significant change in cell morphology [118]. These outcomes thus demonstrate the feasibility and efficiency of using gelatine combined with hASCs to facilitate burn wound repair in the near future. Another study used mouse bone marrow mesenchymal stem cells (MSCs) seeded in a biodegradable scaffold made of arginine-based polyester amide and chitosan in the treatment of third-degree burn wounds in mice [114]. Six days after the seeding, 97% of mice MSCs adhered to the scaffold without compromising cell viability and cytotoxicity. In addition, when applied to the wounded area, MSC seeded scaffolds promoted wound closure of the necrotic skin and excised third degree burn wounds by 41% compared to the control scaffold containing medium only. Moreover, MSC seeded scaffolds yielded larger granulation tissue area and higher vascularity, resulting in improved recovery of the wounded area. Amino acid-based polyester amide is a relatively new class of synthetic biomaterial that possesses biodegradable, biocompatible, and non-toxic behavior. Incorporating chitosan with polyester amide exhibits excellent properties and therefore is a promising candidate for use as the scaffold and delivery carrier of MSCs and wound coverage to treat severe burn wounds [129]. Although previous studies on the direct application of MSCs to the wounded site without scaffolds resulted in apoptosis, in this study, the biomaterial scaffold that carried MSCs demonstrated effectiveness in promoting the critical healing process including wound closure, re-epithelialization, granulation tissue growth, and blood vessel regeneration [114].

## 8. Role of Biomaterials and Stem Cells in Skin Tissue Engineering for Wound Healing and Regeneration Treatment

From the reviewed studies (Table 3 and Table 5), it is evident that both stem cell therapy and stem cell therapy combined with biomaterials show improved wound healing capacity and regeneration to skin injury. However, the healing or correction time for only stem cell-based therapy (Table 3) takes a longer time, from 7 to 28 days, compared to the combined therapy of stem cell and biomaterials (Table 5), which has a time period of 7 to 14 days. The shorter time of the combined therapies can be due to the porous microstructure of the bioscaffold that serves as a 3D microenvironment for stem cell growth and the regulation of growth factors. It essential to provide communication between cells and between cells and the ECM. Thus, the scaffold supports the attachment, proliferation, and differentiation of cells like the in vivo environment; so, when applied to the wounded area in vivo, the stem cells are still viable, and upon recognizing their surrounding microenvironment, they can efficiently contribute to wound healing process and regeneration.

Apart from that, the biomaterials are designed, considering cell delivery materials, including cell survival and retention, the regulation of cell fate, and integration of host tissues to further promote in vivo studies and clinical translation of stem cell therapies [130]. One of the critical problems associated with stem cell therapy is acute death and low retention of delivered cells at the transplant site, which can hinder or delay the wound healing mechanism [131]. Besides, factors such as shear stress, absence of cell adhesive ligands, oxidative stress, inflammation, and vascularization failure can result in cell death and necrosis post-transplantation [132]. In such a scenario, the delivery of the cell via a hydrogel precursor can reduce the mechanical forces experienced during cell delivery [132]. Not only that, but they can also act as protective barriers to prevent attacks from immune cells [133] and prevent cell damage from reactive oxygen species (ROS) as well as insufficient oxygen and nutrients supply upon modification with cell binding peptides, antioxidant ligands, or nanoparticles. [134]. Moreover, the encapsulation and microencapsulation strategy of stem cell aggregates within biomaterials can prevent leakage of cell suspension at the injection site. Furthermore, it enhances the survival and retention of delivered stem cells [135].

The design of biomaterials with controlled biodegradable quality also enhances cell engraftment. To integrate with the host tissues, transported cells should be able to migrate and deposit ECM throughout biomaterial as it degrades [136]. The porous scaffolds with the open interconnected structures provide sufficient volume for cell loading, migration, and proliferation, improving the transplanted cells’ chances for engraftment [137]. Biomaterials also provide biophysical and biochemical factors that facilitate controlled self-renewal and differentiation of stem cells for regeneration. Morphological characteristics and mechanical properties are the most investigated biophysical cues for stem cell-based skin tissue engineering [130]. For instance, the biomaterial’s matrix elasticity and dynamic viscoelasticity can direct the lineage specification of MSCs [138]. Moreover, the structure of biomaterials also affects stem cell fate through guiding cell morphogenesis and alignment. For example, micro and nanopatterned matrices were reported to regulate self-renewal and differentiation of stem cells at both single and multiple cell levels [139]. In addition to biophysical factors, biochemical factors such as growth factors, small molecules, and ECM conjugates have been combined with stem cells to direct stem cell fate that targets specific intracellular pathways and enhance differentiation of stem cells [140]. Furthermore, delivering stem cells using injectable hydrogel matrices such as collagen and gelatine (Table 5), which have abandoned tethered signaling factors, facilitate regeneration of skin tissue resulting in the recovery of the wounded region. Hence, biomaterial-based assemblies of stem cell simplify the cellular interactions that promote morphogenesis and tissue organization similar to that which occurs during embryogenesis, thereby ensuring efficient wound healing and regeneration of the lost tissue. 

Although combined application of stem cells and biomaterials (Table 5) show improved wound healing and regeneration attributes in several in vitro and in vivo studies, most commercially used skin substitutes are manufactured from autologous cells. At present, allogenic, xenogenic, synthetic, and acellular constructs are also gaining considerable attention for clinical translation purposes [141]. However, there are no existing commercial skin constructs available in the market that are constructed using both stem cells and biomaterials. This is because tissue engineering of skin using biomaterials and stem cells is still a growing field in regenerative medicine. Although tremendous efforts are being undertaken in employing micro and nanofabrication strategies, biomaterial synthesis, and stem cell culture and functionalization techniques, there are still challenges involved in terms of characterization, optimization, and delivery of treatment [142]. In addition, there are also unresolved complications such as wound contraction, impaired vascularization, and high costs associated with these products that require careful attention [1]. Besides, the application of cell-based skin substitutes has been limited in clinics due to time-consuming and labor-intensive process and the short life span of the product [142]. Therefore, the translation of such artificial skin to clinics, manufactured with novel technologies incorporating stem cells with biomaterials, requires further predictive test methods and appropriate standards and regulations to ensure its reproducibility, functionality, and reliability on commercial scale.

## 9. Conclusions and Future Perspectives

In summary, stem cells have received attention in skin tissue engineering and regenerative medicine due to their self-renewal ability and capacity to differentiate into specific cell types. However, immune sensitivity, compromised survival, proliferation, and differentiation rate limit the application of stem cells in clinical trials as well as in vitro and in vivo applications. With the aid of biomaterials, these barriers can be overcome. Natural and synthetic biomaterials can be rationally designed for wound healing treatment according to their biophysical and biochemical properties. The incorporation of stem cells into structured and modified biomaterials increases the competence of restoring and repairing dysfunctional skin tissue and promote wound healing parameters such as improved epithelialization, granulation tissue formation, vascularization, and angiogenesis. The well-organized spatial properties of a biomaterial or scaffold, in turn, can provide a protective and sometimes inducible microenvironment for the stem cells, mimicking the natural ECM. In addition, biomaterials are also being used to regulate stem cell fate before and after delivery by providing mechanical and biochemical support. Despite the encouraging results in non-clinical studies, only a handful of biomaterials have been used for stem cell-based therapies in patients. Thus, additional clinical trials that use biomaterial should be performed to elucidate the influence of materials’ biophysical and biochemical properties on wound healing, tissue repair, and regeneration of humans. Hence, we recommend future efforts to improve the clinical outcome in designing and fabricating biomaterials using emerging techniques like 3D bioprinting, electrospinning, and nanotechnology to meet specific properties of the components that need to be delivered for wound healing and regeneration.

## Figures and Tables

**Table 1:** Classification of scaffold fabrication techniques used in skin tissue engineering.

Fabrication Techniques		Advantages	Disadvantages
Conventional fabrication techniques	Electrospinning	Essential for developing nanofibrous scaffolds, homogenous mixtures made of fibres with high tensile strength [24]	Process depends on many variables, problematic to obtain 3D structures with the required pore size needed for biomedical application [26,27]
	Freeze drying	Used in a variety of purposes, capability of obtaining high temperature, manageable pore size by changing freezing method [24]	High energy consumption, long term timescale, generation of irregular size pores [28]
	Gas foaming	Porosity up to 56.71% [29]	Temperature dependent, product obtained from decreased temperature might have closed pore structure or a solid polymeric skin [30]
	Thermal induced phase separation	Porosity up to 80% [31], can use low temperature to integrate bioactive molecules [24]	Only used for polymers amenable to phase separation [31]
Rapid prototyping (RP)	Bioprinting	Low cost, higher accuracy, and greater shape complexity [24]	Depends on the cells/biomaterials used [32]
	Fused deposition modelling (FDM)	High tensile strength [24]	Has limited application to biodegradable polymers [33]
	Solvent based extrusion free forming (SEF)	Used to make ceramic, metal, and metal/ceramic composite part; used for precise control of scaffold structure at the micron level [24]	Variation in temperature affects extrusion pressure, including nozzle length-to-diameter ratio, and the extrusion velocity [34]
	Stereolithography	High resolution, uniformity in pore connectivity [24]	Requires a massive number of monomers and post-polymerization treatment to improve monomer conversion [35,36]

**Table 2:** Function of various GFs and their outcome in wound healing.

Growth Factors	Origin of Secretion	Function	Study	Outcome
bFGF [39,45]	Endothelial cells, macrophages, monocytes	Stimulate proliferation, migration, and angiogenesis	In vivo	Improved re-epithelialization, angiogenesis, and collagen deposition in diabetic mice wound model
EGF [41,44]	Platelets, macrophages, fibroblasts	Epithelialization	In vivo	Enhanced granulated tissue formation, cell migration, and re-epithelialization in diabetic mice wound model
PDGF [42,45]	Platelets, keratinocytes, macrophages, endothelial cells, fibroblasts	Promotes cell proliferation, migration, and angiogenesis	In vivo	Enhanced granulation tissue formation and collagen deposition in full-thickness incision mice wound model
TGFβ_3_ [40,48]	Platelets, keratinocytes, macrophages, lymphocytes, fibroblasts	Inflammation, granulation tissue formation, epithelialization, matrix formation and remodeling	In vivo	Decreased scar formation in a rabbit model by reducing the ratio of type I to type III collagen
VGEF [43,45]	Platelets, macrophages, keratinocytes, endothelial cells	Epithelialization, collagen deposition, angiogenesis	In vivo	Promoted angiogenesis, collagen deposition, macrophage polarization and granulation tissue formation in full-thickness incision mice wound model

**Table 3:** Use of various stem cells in wound therapeutics.

Types of Stem Cells	Wound Types	Mode of Delivery	Correction Time	Model Used	Treatment Effects
Adipose tissue derived MSCs [64]	Excisional wound	Scratch wound assay	14 days	Rat	Accelerate migration and proliferation of fibroblast and keratinocytes
ASCs [67]	Full thickness wound	Transplantation	14 days	Murine	Promote wound healing, reduce scar formation, and minimized long term side effects of cellular transplantation
ASCs [75]	Full thickness wound	Subdermal injection	28 days	Rat	Increased tissue regeneration, suppression of inflammatory response, augmented EGF and VEGF production, promote re- epithelialization and cell infiltration
Autologous MSCs [65]	Cutaneous wound	Subjection	14 days	Mouse	Promote wound repair by regaining wound tensile strength
BMSCs [71]	Excisional wound	Subcutaneous injection	10 days	Mouse	Complete re-epithelialization and wound closure with a prominent keratinized layer
Human iPSCs [72]	Excisional wound	Intradermal injection	14 days	Mouse	Promote angiogenesis, accelerated wound closure, and increased wound perfusion
Human iPSCs [21]	Excisional wound	Intravenous injection	14 days	Mouse	Accelerated epithelialization

**Table 4:** Examples of natural and synthetic biomaterials used in skin tissue engineering along with their pros and cons.

Types	Examples	Advantages	Disadvantages	Major Properties in Wound Healing
Natural biomaterials	Alginate [83,84]	Can retain its shape due to low viscosity and zero shear viscosity	Inert material and only suitable for in vitro assays, requires crosslinking due to low bioactivity	Porous, good absorption, biocompatible and biodegradable nature promote wound healing resulting in less scarring, minimal bacterial infection, and the creation of a moist wound environment
	Cellulose [85,86]	Flexibility in shape, easy processing, good mechanical strength, and biodegradability	Lack of solubility in water and many organic solvents	Hydrophilic nature, purity, ability to maintain appropriate moisture balance and flexibility form a tight barrier between the wound and the environment, preventing bacterial infections
	Chitosan [78,87]	Possess antibacterial, antifungal, mucoadhesive and analgesic property	Poorly soluble in aqueous solutions except for acidic medium	Interact with negatively charged molecules (protein, fatty acid, bile acid, polysaccharide, phospholipids); chelate metal ions (iron, copper, magnesium); stimulate hemostasis and accelerate tissue regeneration
	Collagen [9,78,88]	Suitable mechanical property and biocompatibility	Susceptible to crosslinking and any sterilization procedure	Triple helix conformation of collagen type 1 favour cell adhesion and migration; pore sizes for the 5 and 8 mg/mL collagen type I scaffolds ranged between 126–188 μm promote connective tissue regeneration
	Elastin [78,89]	High elasticity	Poor mechanical strength and availability	Half-life > 70 years and the monomer can reversibly stretch up to eight times its resting length; fibre alignment positively affects cell phenotype, adhesion, and proliferation
	Fibrin [78,90]	Good protein binding ability that promotes vascularization	Limited control over its structural and mechanical properties	Fibrin network serves as a provisional template for promoting cell migration and proliferation; releases cytokines and growth factors attracting inflammatory cells at the wound bed; activates re-epithelialization, angiogenesis, connective tissue formation and contraction
	Gelatin [2,82,91]	Low antigenicity and higher solubility in solvents	Lack high mechanical resistance	Porous gelatin matrices absorb wound exudates, maintain a moist environment essential for wound healing
	Silk fibroin [83,92]	Biocompatible with strong mechanical properties	High brittleness	Porous template supports cell proliferation, differentiation, and ECM production
Synthetic biomaterials	PCL [93,94]	Biocompatible with relatively slow degradation time	Poor cell attachment due to hydrophobicity	Show desirable electroactivity, biocompatibility, free radical scavenging capacity and antibacterial activity; promoted collagen deposition and granulation tissue thickness during the process of wound healing
	PEG [9,78,95]	Reasonable control over structural and compositional properties	Lacks interactive cell character	Demonstrate biocompatible property, protein resistance, non-immunogenicity, non-toxicity, and good water solubility required for chronic wound healing
	PGA [9,78,96]	Highly biocompatible and biodegradable	Rapid mechanical strength loss	Exhibit reasonable wetting time, preferable surface morphology, low moisture uptake and prolonged swelling behavior
	PHA [97,98,99]	Low acidity and bioactivity, nontoxic degradation, biocompatibility, and non-carcinogenicity	Poor mechanical properties, high production cost, limited functionalities, incompatibility with conventional thermal processing techniques	Structural porosity and wettability similar to natural ECM, effectively promoting cellular migration, attachment, and proliferation
	PLA [96,100]	Easy modification with other biomaterials and bioactive compounds	Poor cell interaction, low elongation, and hydrophobicity	Exhibit high mechanical properties, reasonable wetting time, preferable surface morphology, low moisture uptake, prolonged swelling behavior and strong antibacterial properties against *Staphylococcus aureus* and *Escherichia coli*
	PLGA [9,78,101]	Biocompatible and biodegradable with a wide range of erosion time	Generates adverse inflammatory reaction upon degradation	Exhibit cytocompatibility and facilitate cell adhesion, spreading and proliferation, release anti-inflammatory factors required for wound healing accelerate collagen deposition and re-epithelialization

**Table 5:** Combined application of biomaterials with stem cells in various wound healing treatments.

Biomaterials Used	Fabrication Method	Stem Cells	Application	Correction Time	Treatment Outcome
Chitosan & arginine based polyester amide [114]	Gel	MSC	3rd degree burn wounds in a mouse model	7 days	Promoted wound closure, re-epithelialization, granulation tissue growth, and blood vessel regeneration
Collagen [115]	Scaffold	hiPSC-SMC	Full-thickness cutaneous diabetic mouse wound	7 days	Increased cellular proliferation, expression of pro-angiogenic and regenerative cytokines and angiogenesis
Collagen with stromal-derived factor-1 alpha (SDF-1α) gene [116]	Scaffold	ADSC	A non-healing diabetic foot ulcer	14 days	Restore the pro-angiogenic regenerative response in the human diabetic ADSCs and exhibited active-matrix remodelling of fibronectin and basement membrane protein collagen IV
Fibrin [117]	Gel	ASC	Rat skins burn model	7 days	Enhanced local angiogenesis of regenerating burn wound without impeding wound closure kinetics up to 21 days, integrates with wound surface allowing ASC transmigration into the regenerating wound and enhanced granulation tissue formation.
Gelatine [118]	Hydrogel	ASC	Murine burn model	14 days	Highest wound contraction rate of 55.3%, decreased discoloration rating, roughness score and reduced scab formation
PCL [119]	Nanofibrous scaffold	BMSC	Full-thickness excisional wound in diabetic mouse	7 days	Enhanced granulation tissue formation, angiogenesis, ECM deposition and elicited pro-regenerative response to accelerate wound healing
PEG [120]	Hydrogel	ADSC	Full-thickness excisional wound in the diabetic rat model	7 days	Inhibit inflammation, promote angiogenesis and re-epithelialization
PLGA [121]	Nanofibrous scaffold	hASC	Full thickness excisional wound in mouse model	7 days	Better cell activity in the PLGA matrix in terms of cell adhesion, proliferation, and survival along with improved wound healing

## Data Availability

The data presented in this study are available on request from the corresponding author.
